# Machine learning model for fast prediction and uncertainty quantification of needle deflection during prostate biopsy

**DOI:** 10.1002/mp.70314

**Published:** 2026-01-31

**Authors:** Nathan Hoffman, Lidia Al‐Zogbi, Axel Krieger, Junichi Tokuda, Pedro Moreira, Mark Fuge

**Affiliations:** ^1^ Department of Mechanical Engineering University of Maryland College Park Maryland USA; ^2^ Department of Mechanical Engineering Johns Hopkins University Baltimore Maryland USA; ^3^ Department of Radiology, Harvard Medical School Brigham and Women's Hospital Boston Massachusetts USA; ^4^ Department of Mechanical and Process Engineering ETH Zürich Zürich Switzerland

**Keywords:** Machine Learning, Mechanics Based Model, Needle Deflection, Prostate Biopsy, Uncertainty Quantification

## Abstract

**Background:**

Accurate needle placement is essential for prostate biopsy. Recently, transperineal prostate biopsies are receiving renewed interest due to concern over infection from conventional transrectal biopsies. However, accurate needle placement is more challenging in the transperineal approach than in the transrectal approach due to the long insertion distance leading to a large targeting error and repeated insertion attempts. Improved procedure planning tools that can predict the deviation of the needle can potentially reduce the targeting error and number of insertion attempts. Prediction of deflection magnitude requires a model of biopsy needle deflection, which in turn requires information about tissue material properties. However, material properties of tissue in patients cannot be easily obtained. Accounting for this uncertainty in patient tissue properties requires a model capable of quantifying uncertainty in needle deflection as a function of a distribution of tissue properties. A Monte Carlo uncertainty quantification requires 1000s of samples, but it is not possible to obtain this many samples in a short enough time for intraoperative procedure planning using published needle deflection prediction models.

**Purpose:**

This work seeks to develop a model of needle deflection fast enough for use in intraoperative procedure planning, validate this model against experimental results, and integrate it into a Monte Carlo uncertainty quantification model.

**Methods:**

This work used a mechanics‐based model of biopsy needle deflection to train a Fourier feature neural network (FFNN) model in order to make predictions with a low computational cost. Both models were validated against experimental data. The neural network model was used in a Monte Carlo uncertainty quantification model to quantify uncertainty in needle deflection arising from uncertain tissue mechanical properties.

**Results:**

This work (1) implemented a mechanics‐based model and a FFNN model. Both models were validated against previously published experiments carried out with tissue phantoms. Both models showed close agreement with the experimental data. (2) We showed that our FFNN model was more accurate than a baseline ordinary least squares model, introducing only about 0.3‐mm tip deflection error compared to the mechanics‐based model. We also showed that our FFNN model makes unbiased predictions with respect to the amount of deflection. (3) We demonstrated a Monte Carlo uncertainty quantification model of needle deflection with a low computational cost of about 20 CPU s. We used our uncertainty quantification model to show how the depth, stiffness, and magnitude of uncertainty in a layer of tissue affect needle deflection. In addition, we showed a simple clinical example of the use of our model.

**Conclusions:**

This work demonstrates a Monte Carlo uncertainty quantification model of needle deflection with a low computational cost. This method shows promise for future applications in procedure planning for prostate biopsies as well as other transperineal procedures conducted with flexible needles such as cryoablation and brachytherapy.

## 1 INTRODUCTION

Prostate biopsies are a common procedure to confirm prostate cancer, with more than one million performed every year in the United States and Europe.^1^ While systematic, unguided prostate biopsy has been commonly performed, targeted prostate biopsy has attracted attention recently as they have been shown to be more likely to detect high‐risk cancers than unguided biopsies.^2^, ^3^, ^4^ This shift is driven by advancements in image guidance technologies, such as in‐bore MRI guidance and MR–ultrasound fusion guidance, and the wide adoption of multiparametric MRI and PI‐RADS reporting, which allow clinicians to detect potential lesions and evaluate their risks more precisely before biopsy.

Accurate targeting is crucial for the successful detection of clinically significant cancers; a simulation study^5^ estimated that targeted biopsies outperform 12‐core systematic biopsies in terms of cancer sensitivity, when the targeting error is less than 3 mm. If subregion sampling is considered, the required accuracy should be substantially smaller than 3 mm; assuming that the target subregion is 50% of the tumor foci by volume,^6^ the needle placement error needs to be less than 2.1 mm.

Despite advancements in guidance technologies, accurately deploying a biopsy needle into a suspected lesion remains challenging. Prostate biopsies use thin, long needles (typically 18‐gauge, 150–200 mm), which can deflect unpredictably in tissue due to anatomical variations, differences in tissue properties, and the complexity of needle–tissue interactions. This challenge is highlighted by the increasing use of the transperineal approach, which addresses infection concerns associated with the conventional transrectal approach.^7^ The transperineal approach generally requires deeper needle insertion than the transrectal approach, often resulting in a large deflection and subsequent repeated reinsertions until reaching the desired foci. As a result, transperineal prostate biopsies have a long learning curve and take longer to perform than transrectal biopsies.^8^

During transperineal prostate biopsy, physicians typically assume a straight needle trajectory and utilize a grid‐based template to guide insertion toward the intended target. However, needle–tissue interactions often induce deviations, preventing the needle from following a perfectly linear path. As a result, multiple insertions are frequently required to accurately reach the target. Integrating needle deflection prediction into procedure planning has the potential to optimize procedural efficiency by minimizing the number of insertions required to accurately sample the targeted region, thereby reducing overall procedure time. To accomplish this objective, a procedure planning tool must include a needle trajectory simulation that accurately estimates needle deflection while maintaining low computational costs to complete the planning process quickly during the procedure.

Many previous publications have presented mechanics‐based needle deflection models which are accurate under laboratory conditions and run within a few seconds, but these models have not been adopted for procedure planning. A major obstacle to their adoption is the impracticality of accurately measuring patient‐specific tissue properties, which can vary greatly due to factors such as age,^9^ the presence and progression of cancer,^10^ or radiation fibrosis.^11^ This variability necessitates an uncertainty quantification model capable of producing a probability distribution of needle paths based on various tissue property scenarios.

In the context of prostate biopsies, uncertainty quantification is often used in state estimation models to estimate uncertainty in the prediction of the next state. Asadian et al. perform a simple type of uncertainty quantification with a mechanics‐based model where they show how varying model inputs ±20% varies the model outputs.^26^ The uncertainty quantification approach we pursue differs from these approaches as we use a Monte Carlo model to quantify the error of an estimate of final needle shape based only on a prior distribution of tissue properties.

In order to estimate a posterior distribution, a Monte Carlo uncertainty quantification model requires a large number of samples. In our case with several dimensions of tissue parameters and a complex needle deflection process, we estimate that thousands of samples are required. Therefore, an uncertainty quantification model for intraoperative procedure planning would require thousands of needle deflections to be predicted within a few seconds. The runtime of about 1 s reported by Wang et al. for their mechanics‐based model would produce a runtime of several minutes to several hours to model thousands of insertions.^12^ For this reason, we are motivated to develop a machine learning (ML) model to predict the magnitude of needle deflection as a function of tissue properties, which is much faster than previously published mechanics‐based models.

Needle deflection in tissue is challenging to simulate due to many intrinsic factors, including the complex physics of tissue deformation, difficulty in obtaining ground truth measurements, and the complexity of needle–tissue interaction. Three different approaches to the prediction of needle deflection in tissue have been popular in published works: kinematic models,^13^, ^14^, ^15^ finite element analysis (FEA) models,^17^, ^18^, ^19^, ^20^ and mechanics‐based models.^12^, ^16^, ^22^, ^24^, ^25^

To generate data for training a ML model, this work uses a mechanics‐based model. Mechanics‐based models are simplified models of needle deflection which model the needle as a beam, and model the reaction from the tissue as a series of springs attached to the needle. Several published mechanics‐based models have been successfully validated against needle deflection in tissue phantoms and tissue samples.^12^, ^16^, ^22^, ^23^, ^24^, ^25^, ^26^, ^27^ We choose to use a mechanics‐based model in this work as they are computationally inexpensive and capable of modeling multiple layers of tissue.

To the best of our knowledge, the only previously published work using a ML model to predict needle deflection independent of sensor input is Behera et al., which trains a decision tree model on needle shapes in polyvinyl alcohol (PVA) tissue phantoms. Their model makes predictions with inputs of insertion depth, phantom PVA concentration, and needle properties to predict needle deflection.^32^ This model is limited as it is only trained on two different homogeneous tissue phantoms.

This paper contributes the following:

1. A ML model for the prediction of needle deflection and a validation of this model with a comparison to needle insertions in complex PVC tissue phantoms. A small number of published works present ML models for predicting needle deflection, but these models are limited by the scope of their training data and the scope of their predictions. We overcome these limitations by training our ML model to predict the results of a mechanics‐based model. Additionally, our ML model makes predictions several orders of magnitude faster than mechanics‐based models.
2. An uncertainty quantification model built on the ML model. Several published works examine state estimation uncertainty quantification, while our model simulates the entire needle insertion process. Uncertainty quantification over the entire insertion is necessary for procedure planning due to the uncertainty in the mechanical properties of tissue.
3. Insights drawn from the uncertainty quantification model regarding how the degree of uncertainty in tissue properties, tissue elastic modulus, and position of tissues affects uncertainty in needle deflection. The greater scope of our uncertainty quantification model allows us to draw insights not available from state estimation models such as the effects of the positions of tissue layers on uncertainty.

## 2 METHODS

We first established a mechanics‐based model capable of modeling the deflection of a needle as the needle is inserted through up to 10 layers of linear elastic isotropic tissue. Next, we used this mechanics‐based model to train several ML surrogate models and evaluated the difference between the predictions of the surrogates and the mechanics‐based model. Finally, we used an ML model to quantify uncertainty under different distributions of tissue properties.

### 2.1 Mechanics‐based model

Our mechanics‐based model used a beam FEA model to simulate the needle, with reaction from the tissue modeled by a series of springs attached to the beam. We choose to use an FEA model for the needle as the alternative, beam theory, can have substantial error for large deflections.^22^ We modeled an 18 gauge needle made out of nitinol, with an elastic modulus of 35 000 MPa. The needle was modeled as a tube with outer diameter 1.27 mm, inner diameter 0.838 mm, and a bevel tip angle of 30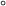. Deflection of the needle develops due to a cutting force applied to the tip of the needle. We discretized the 140‐mm needle into 47 beam elements (48 nodes) and calculated the shape of the needle using FEA. Our FEA simulation was performed using the PyniteFEA Python library version 0.88. To calculate cutting force at the tip, this work followed the approach of Liu et al.,^22^, ^23^ which assumed that the bevel tip compresses the tissue between each insertion depth discretization and used this to calculate a reaction force on the tip. Liu et al. additionally simplified the bevel tip to a wedge. The cutting force $F_{c}$ was calculated as follows:

(1) $$F_{c}=\frac{C_{\text{tip}\;}d^{2}}{4\tan(\alpha /2)}$$

**FIGURE 1:**
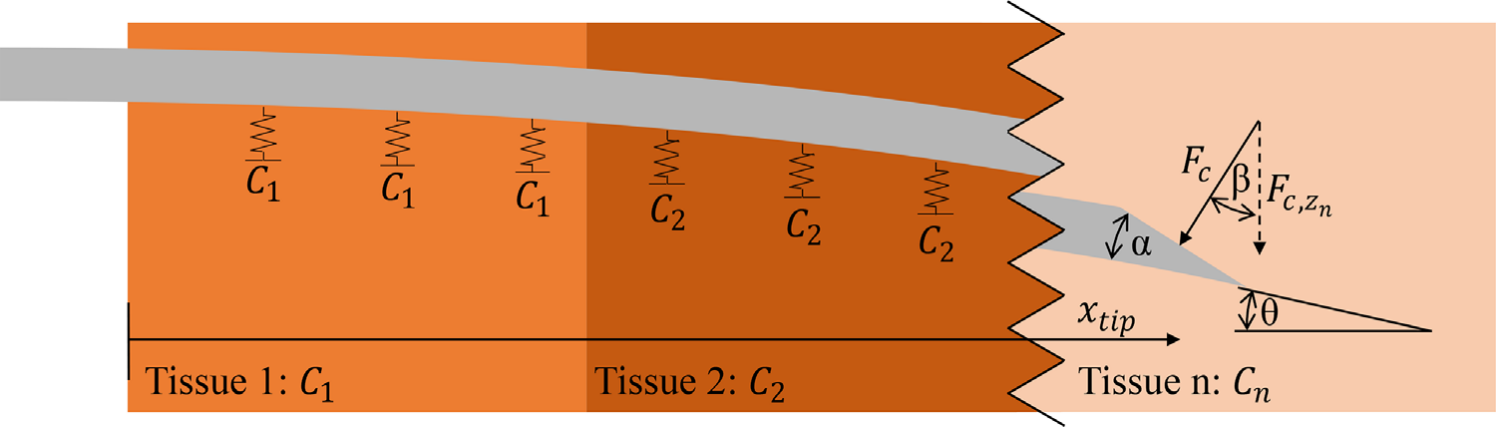
Graphical representation of the forces in the mechanics‐based model.

Here, $C_{\text{tip}\;}$ is the elastic modulus of the tissue at the tip, d is the diameter of the needle, and α is the angle of the needle bevel tip, as shown in Figure 1. The force that causes the needle to deflect is the *z* component of the cutting force, $F_{c,z_{n}}$:

(2) $$F_{c,z_{n}}=F_{c}\cos\left(\beta \right)$$

(3) $$\beta =\alpha +\theta \left(x_{\text{tip}\;}\right)$$

where $\theta \left(x_{\text{tip}\;}\right)$ is the slope of the needle at the tip, and β is the angle between $F_{c}$ and the direction of deflection.

In our mechanics‐based model, we defined tissue properties for every millimeter of depth, and inserted the needle by 1 mm for each step of the simulation. Our mechanics‐based model worked as follows: (Step 1) a spring of the corresponding tissue elastic modulus was attached to the node behind the needle tip (on the first step there are no springs attached as the needle is outside the tissue); (Step 2) the cutting force was calculated and applied to the tip of the needle; (Step 3) the deflection of the needle was calculated by running the FEA model; (Step 4) every 3 mm (as each beam element is 3‐mm long) of insertion the previously added springs were moved (while preserving the compression or tension on the spring) back towards the needle base. This process was repeated until the needle tip reached the desired depth.

Our mechanics‐based model used linear elastic isotropic material properties to model the tissue. To generate training data for the ML model, 50 000 insertions were simulated in the mechanics‐based model. This data was split into a set of 10 000 insertions for training and hyperparameter tuning, and 40 000 for validation. Each simulation had 1–10 layers of tissue. The number of layers and the transition depths between layers were sampled uniformly. Within each layer, the tissue properties were homogeneous. The Young's modulus of each layer was uniformly sampled between 0 and 0.2 MPa.

The ML problem was defined as follows: The model input was a vector of 126 dimensions with 125 dimensions corresponding to tissue elastic modulus with one sample every millimeter of depth and one additional dimension representing the depth the needle is inserted to. The prediction target was a vector of 48 dimensions, where each dimension corresponded to the deflection of one beam nodes from the FEA model.

The Fourier feature neural network (FFNN) in this work adopted the approach of Tancik et al. This approach was as follows: Starting with an input $x\in R^{d}$, create a matrix $B\in R^{m\times d}$ where $B_{i,j}∼N\left(0,1\right)\times s$. s is a scaling hyperparamter and m is a hyperparameter controlling the number of Fourier features created for each dimension of the input. The input feature vector, x, is then transformed as follows:

(4) $$x_{\text{proj}}=2\pi xB^{T}$$

The Fourier feature mapping is then applied to $x_{\text{proj}}$:

(5) $$x_{\text{map}}=\left[sin\left(x_{\text{proj}}\right),cos\left(x_{\text{proj}}\right)\right]$$

The result, $x_{\text{map}}$, was used as the input of a neural network which, in the case of this work, was a multilayer perceptron (MLP).

### 2.2 Surrogate models

Surrogate models are an engineering method where a model with low computational cost is used to predict the output of a model with high computational cost. To create a baseline surrogate model, an ordinary least squares (OLS) linear regression was trained. This OLS model was trained on 1 000 needle trajectories at 124 insertion depths for a total of 124 000 training samples. Additional samples in the training set were not used to train the OLS regression due to computational cost and lack of improvement in performance. We used a set of 40 000 needle trajectories to validate the OLS model and all other models.

Next, a MLP regressor with two hidden layers of 48 neurons was trained and tested on the same dataset as used with the OLS model. The MLP regressor used the implementation from scikit‐learn 1.3.0 with all hyperparameters besides hidden layer sizes set to defaults. To normalize the dataset, we apply *z*‐score standardization. Finally, we trained a FFNN, which is an MLP regressor augmented with a Fourier feature transformation. One dimension of Fourier feature was used for each dimension of input. One of the effects of the Fourier transform is to normalize the input data (as the output of a sine function is between −1 and 1). For the outputs, we use *z*‐score standardization. Training and inference for the FFNN was performed using PyTorch 2.0.1 and CUDA 11.7. This model was trained with an Adam optimizer. A hyperparameter grid search was performed on learning rate and the Gaussian scale of the Fourier feature mappings. The learning rate was sampled at 10 levels in a log space between 1e‐2 and 1e‐5, and the Gaussian scale parameter was sampled at 10 levels in a linear space between 0.01 and 1. A batch size equal to the size of the training set (10 000 insertions) was used for 500 training epochs.

### 2.3 Experimental validation

**FIGURE 2:**
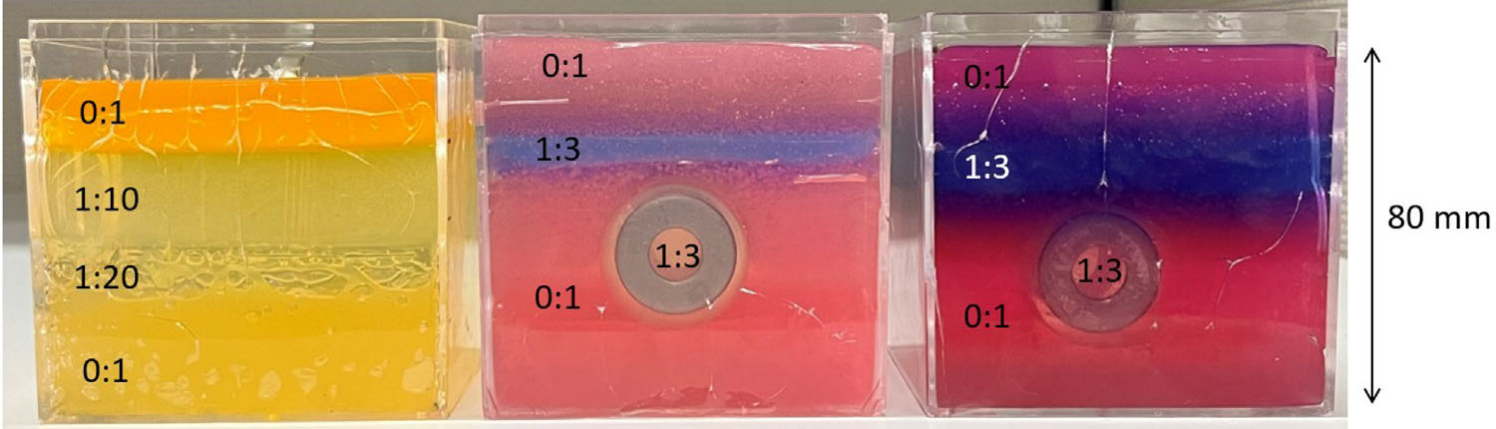
Structure of phantoms used for validation. Needles are inserted from the top, in the direction of the bottom of the page. The ratios shown are for hardener to plastisol volume. From left to right, multilayer phantoms, phantom with Cylinder 2, phantom with Cylinder 3.

We compared the predictions of our FFNN model to results obtained from needles inserted into PVC tissue phantoms. The experimental results used in this work were previously published and used to validate a different mechanics‐based model by Wang et al. The experimental data were obtained from two examples of three types of phantoms (five phantoms total), shown in Figure 2. Wang et al. segment the needle shape from CT scans from a Brainlab Loop‐X taken at a voxel size 0.46 × 0.46 × 0.46 mm^3^.

These phantoms' tissue properties were manually fitted to obtain agreement with the experimental data, which were collected by segmenting CT scans of a needle inserted into a tissue phantom. We achieved agreement between the neural network and the experimental data to within 1 mm in almost all cases. For details on these experiments and the preparation of the phantoms, see Wang et al.

### 2.4 Uncertainty quantification model

Our uncertainty quantification model was a Markov chain Monte Carlo model created using the PyMC Python library . While direct sampling could have been used for the example presented in this work, as the tissue properties are assumed to be independent, Markov Chain sampling facilitates incorporating nonindependent tissue properties. For the purpose of evaluating our uncertainty quantification model, we created a simplified anatomy composed of two tissues as follows: one tissue forming a thin layer and the rest of the tissue being a different type. The position of the thin layer and the properties of both tissues are varied through our experiments. Each of these tissues was modeled with linear elastic properties sampled from a truncated normal distribution. The truncated normal distribution had a minimum of 0 to ensure that no tissue has negative elastic modulus. Needle deflection uncertainty under this tissue elastic modulus prior was determined by first sampling from the tissue property distributions and then using this set of sampled properties to obtain deflection predictions from the FFNN surrogate.

We also show a clinical example of our uncertainty quantification model. In this example, we ran our model on an anatomy obtained from segmenting an MRI scan of a prostate biopsy patient in a robot‐assisted MRI‐guided prostate biopsy study. This biopsy was performed with an 18 gauge biopsy needle (18‐gauge Fully Automatic Biopsy Gun, 150 or 175 mm, Invivo Corporation, Gainesville, FL). Further details on this study may be found in the original publication. The tissue and final needle shape were manually segmented in 3DSlicer from an MRI image obtained during the procedure with a 3‐T wide‐bore (70 cm) MRI scanner (MAGNETOM Verio, Siemens AG, Erlangen, Germany). We compared the prediction of the model to the actual shape of a biopsy needle during this procedure. To obtain a prior for tissue properties, we conducted a search of the literature for measurements of elastic modulus for human muscle and prostate tissues. Kelly et al. have published a review of published prostate tissue elastic moduli and found a mean of 17.5 kPa for benign prostate tissue, and 70 kPa for cancerous prostate tissue^9^. We therefore averaged these values together (to make the model unbiased with respect to cancer), and used a mean of 43 kPa for our model. Kelly et al. did not report a standard deviation across all studies, so we used a standard deviation of 80 kPa in our model to capture the wide range of reported values that Kelly et al. found. We could find no published measurements of pelvic diaphragm elastic modulus in compression, so we instead used a measurement of human gastrocnemius elastic modulus from Mo et al. As the properties of muscle tissue vary depending on direction, we used the average reported across 0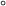, 45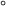, and 90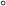 orientations. Mo et al. reported parameters for a one term Ogden model, so we used the equation for tangent modulus at zero compression. This gave a tangent modulus of 10.3 kPa. Mo et al. did not report a value we could easily convert to a standard deviation (as the error varies as a function of the strain), used a small sample size, and tested a different muscle than we are modeling, so we choose a large standard deviation of 80 kPa.

## 3 RESULTS

### 3.1 Surrogate models

**FIGURE 3:**
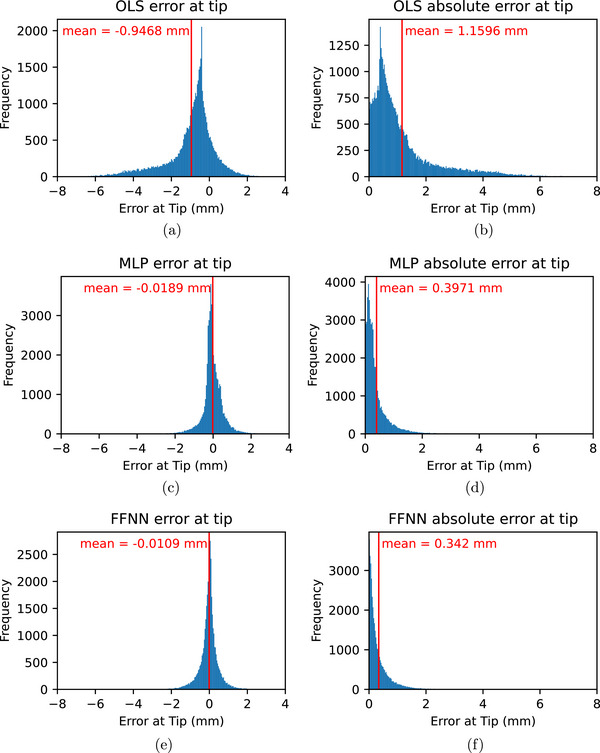
Comparison of error and MAE distributions for OLS, MLP, and FFNN models at 65‐mm insertion depth. (a) Error distribution of OLS model. (b) MAE of OLS model. (c) Error distribution of MLP model. (d) MAE of MLP model. (e) Error distribution of FFNN model. (f) MAE of FFNN model.

We evaluated the OLS model, and all other surrogate models, at an insertion depth of 65 mm as this is a typical insertion depth for a transperineal prostate biopsy. The OLS model achieved an MAE of 1.15 mm at 65‐mm depth, as shown in Figure 3a. We also observed that the error distribution of the OLS model was nonsymmetric about the mean and had a nonzero mean, as shown in Figure 3b.

We observed that the MLP performance began to saturate at a network architecture of two layers of 48 neurons. This MLP model outperformed the OLS regression, with about 0.42‐mm MAE at 65‐mm depth, shown in Figure 3d. This was better than the OLS model. We also observed that the error distribution was more symmetric and had a mean close to zero, as shown in Figure 3c. The mean of the error being closer to zero at specific depths indicated that the MLP does not bias predictions depending on the depth, which the OLS model does. In addition, the more symmetric distribution of the error indicated that the MLP was better able to capture the nonlinear features of the dataset.

Our hyperparameter grid search for the FFNN determined that a Gaussian scale of 0.2 and an initial learning rate of 0.005 achieved the best results. These parameters achieved an MAE at 65‐mm depth of 0.34 mm as shown in Figure 3f. We also observed in Figure 3e that the FFNN produces a prediction error closer to zero than the MLP model, which indicates that the predictions of the FFNN were less biased than the predictions of the MLP.

**FIGURE 4:**
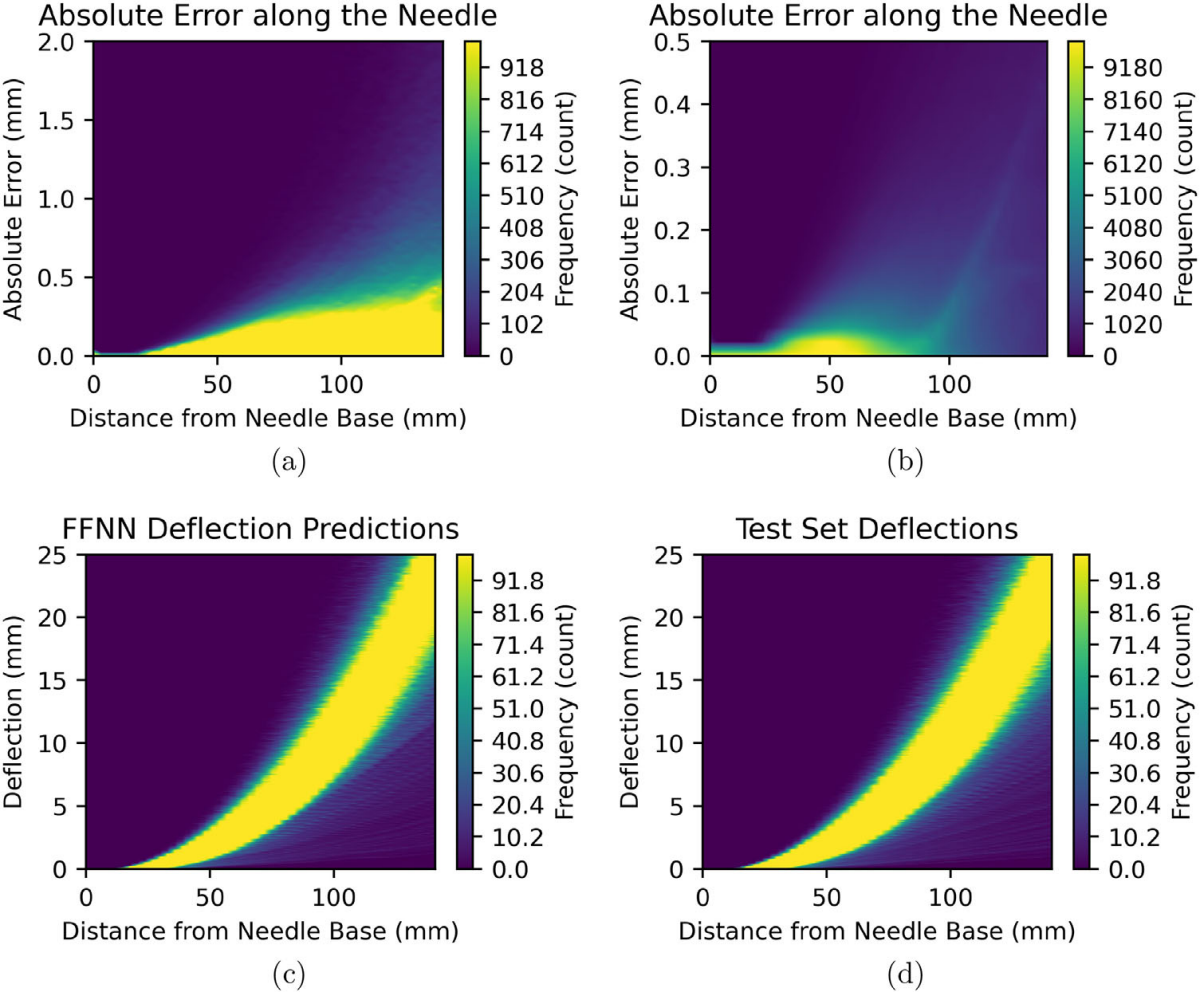
(a) Distribution of absolute error along the length of the needle for all 120‐mm insertions in the test set. Bins above 1000 are clipped to 1000. (b) Distribution of absolute error along the length of the needle for all 120‐mm insertions in the test set. Bins above 10 000 are clipped to 10 000 for visibility reasons. Note that this is the same data as (a) but with a different color‐mapping and range. (c) Distribution of needle deflections predicted by the FFNN along the length of the needle for 120‐mm insertions in the test set. Bins above 100 are clipped to 100. (d) Distribution of needle deflections in the test set along the length of the needle for 120‐mm insertions in the test set. Bins above 100 are clipped to 100.

The error between the FFNN and the mechanics‐based model over the entire length of the needle is shown in Figures 4a and 4b. As some bins near the base of the needle contain a large portion of the samples, we clip the bins above 1000–1000 in Figure 4a and above 10 000 to 10 000 in Figure 4b so that the features of the distribution are visible. For the first 20 mm from the base of the needle, the needle was outside of the tissue, so the deflection was modeled to be 0. We observed that the surrogate has very low error in this region, which is likely because the neural network readily trained towards this constant prediction. For 20–60 mm, we observed that error slowly increases. The stepped widening of the yellow region of the error distribution seen between 20 and 50‐mm distance is an artifact of the chosen bin size of 0.01 mm. Between 60 and 80‐mm distance, we observed that the widening of the yellow region accelerates.

Above 100 mm, we observed that the distribution becomes multimodal as is visible in Figure 4b. We also observed several small modes between 1 and 2‐mm absolute error in Figure 3d, but as the height of these small modes is much smaller than the height of the whole distribution, these small modes may be noise. To further investigate this phenomenon, we plot the distribution of needle deflections predicted by the FFNN along the length of the needle for all deepest insertion states in the test set in Figure 4c. We also plot the distribution of the test set deflections in Figure 4d. We observed that the distribution of predicted and target deflections for each distance above 100 mm is not smooth, but had several sudden changes in frequency. Note that the step‐like features observed as the distance from needle base increases are due to the needle being discretized into 48 nodes.

### 3.2 Experimental validation

**FIGURE 5:**
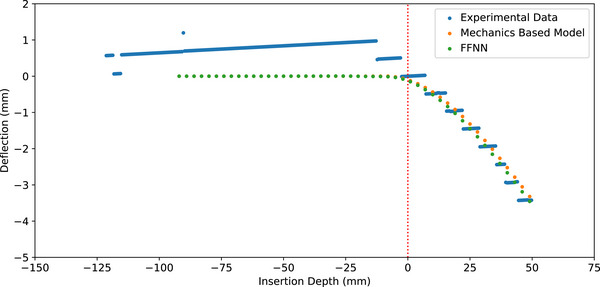
Comparison between data obtained from experimental results, the mechanics‐based model prediction, and the neural network surrogate prediction.

The material properties obtained after manually fitting the mechanics‐based model and FFNN to the data are shown in Table 1. Table 2 shows the mean absolute difference between the FFNN and experiments, mechanics‐based model and experiments, and the FFNN and the mechanics‐based model. A comparison between the FFNN, mechanics‐based model, and experimental data is shown for a single case in Figure 5. As we were only able to test each insertion scenario once, we are unable to calculate a confidence interval for the experimental results. While we are able to establish confidence intervals for our model in the clinical example by using published measurements of tissue mechanical properties to establish a prior distribution for input to our deflection prediction model, the phantom study uses a different approach. In the phantom study, we fit the deflection prediction model to the results, so we cannot use the uncertainty quantification model to obtain a confidence interval.

**TABLE 1:** Manually fitted material properties for phantoms.

	Elastic modulus (MPa)			
Phantom	Layer 1	Layer 2	Layer 3	Layer 4
Phantom with Cylinder 1	0.1	0.05	0.1	0.05
Phantom with Cylinder 2	0.03	0.015	0.03	0.015
Phantom with Cylinder 2 (2nd trial)	0.03	0.015	0.03	0.015
Phantom with Cylinder 3	0.009	0.0045	0.009	0.0045
Multilayer Phantom 1	0.028	0.014	0.028	N/A
Multilayer Phantom 2	0.029	0.0145	0.029	N/A

**TABLE 2:** Summary of experimental validation.

		Mean absolute difference at tip (mm)		
Phantom	Number of insertions	FFNN to data	Mechanics‐based model to data	FFNN to mechanics‐based model
Phantom with Cylinder 1	16	0.3	0.246	0.094
Phantom with Cylinder 2	9	0.3	0.128	0.087
Phantom with Cylinder 2 (2nd trial)	6	0.2	0.250	0.025
Phantom with Cylinder 3	6	0.3	0.238	0.183
Multilayer Phantom 1	4	0.1	0.136	0.111
Multilayer Phantom 2	4	0.2	0.195	0.059

**TABLE 3:** Parameters of normal distributions of tissue elastic moduli in Figure 6.

Subplot	Color	μ (MPa)	σ (MPa)
a	Blue	0.005	0.0005
	Yellow	0.1	0.05
b	Blue	0.005	0.0005
	Yellow	0.1	0.05
c	Blue	0.005	0.0005
	Yellow	0.1	0.05
d	Blue	0.005	0.0005
e	Blue	0.05	0.0005
	Yellow	0.1	0.05
f	Blue	0.05	0.0005
	Yellow	0.1	0.05
g	Blue	0.05	0.0005
	Yellow	0.1	0.05
h	Blue	0.05	0.0005

### 3.3 Uncertainty quantification model

**FIGURE 6:**
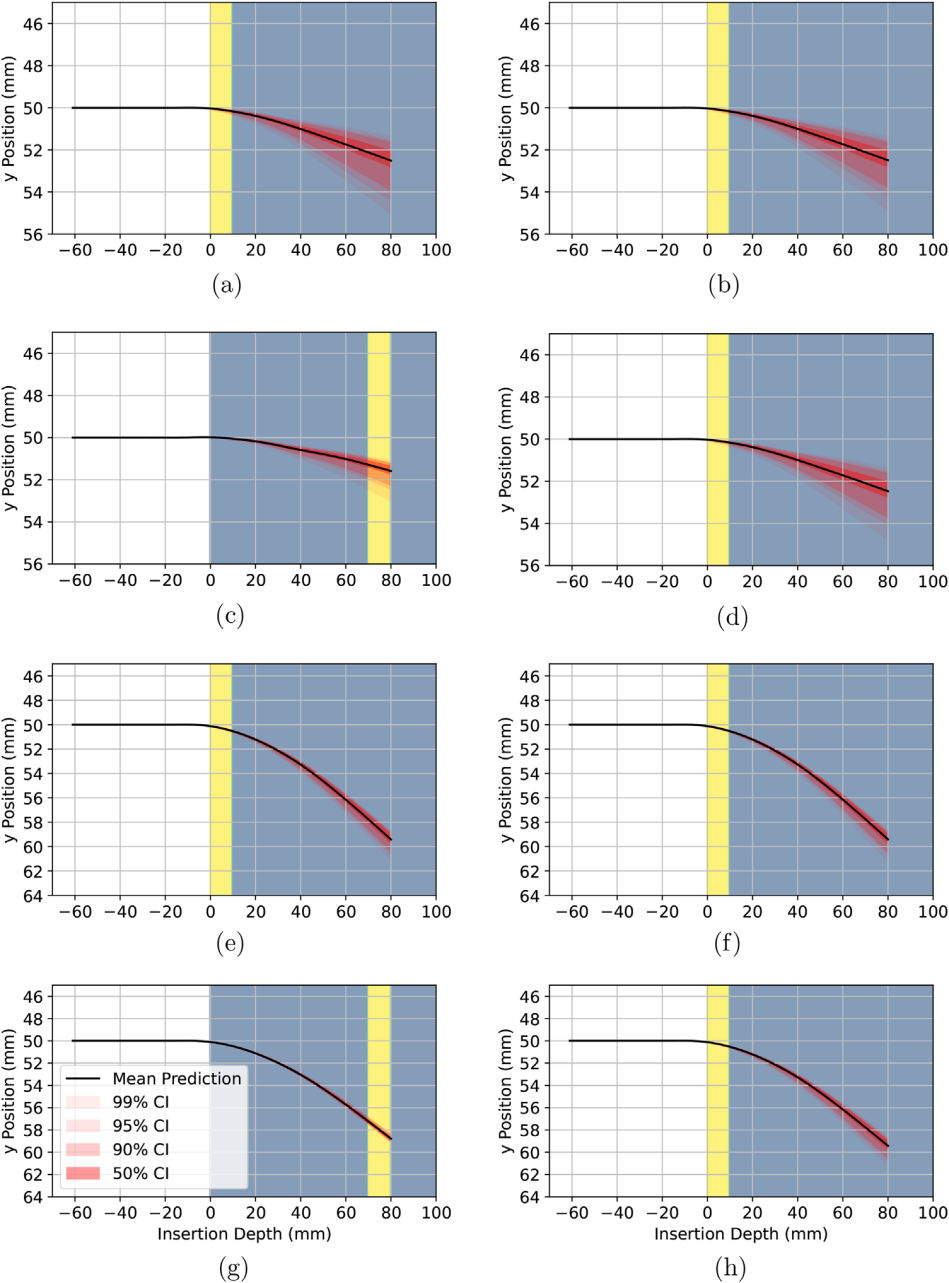
Uncertainty quantification experiments performed for different tissue elastic modulus distributions and anatomies. The thin yellow layer of tissue is always stiffer than the blue tissue. See Table 3 for the tissue elastic modulus distributions used in each example.

**FIGURE 7:**
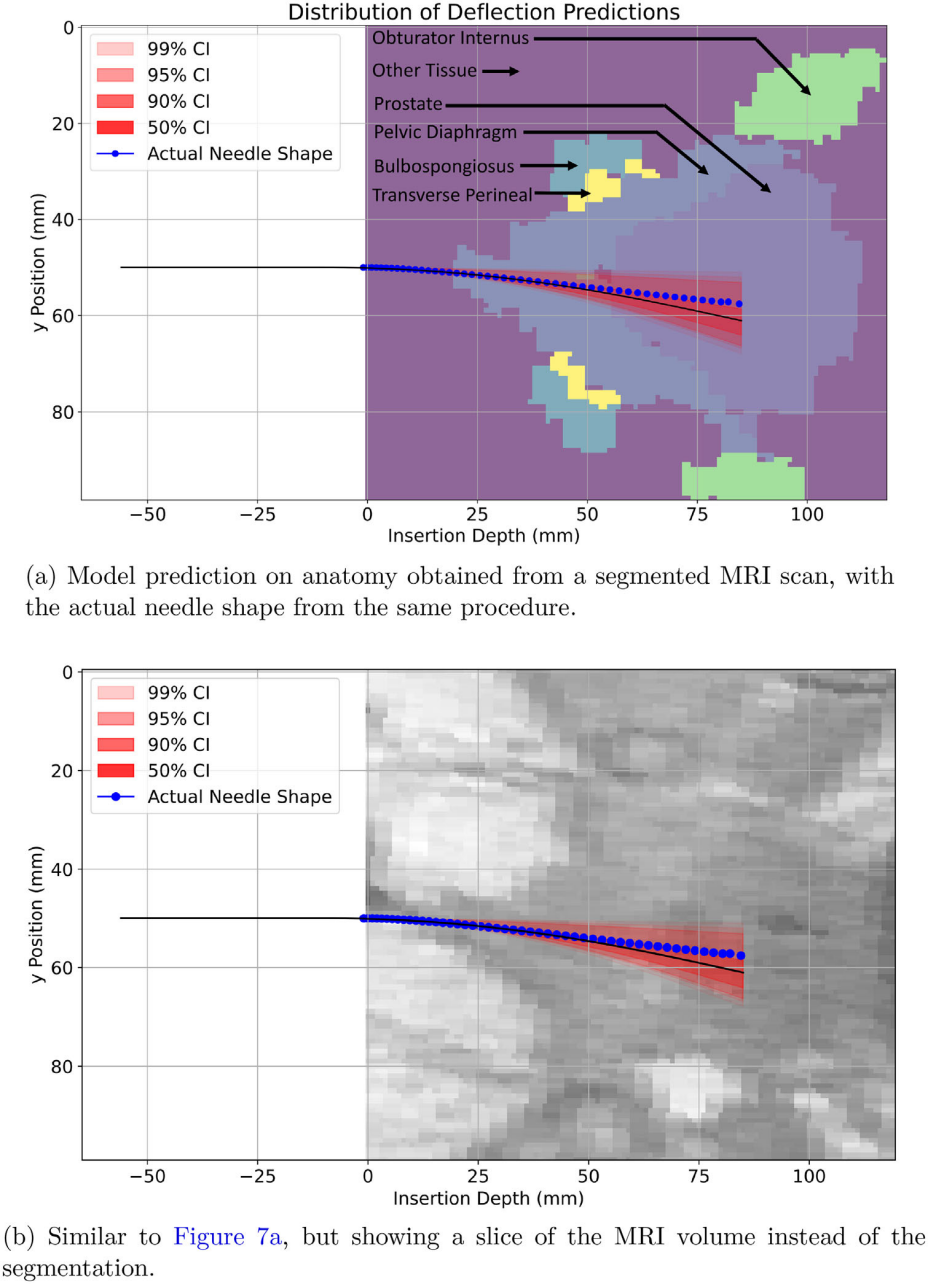
Segmented (a) and raw MRI image (b) for the same anatomy with corresponding model predictions and actual needle shapes.

The proposed uncertainty quantification model took 10–20 s to run depending on the complexity of the anatomy and the depth of the needle insertion. The anatomies and final needle shape confidence intervals are plotted in Figure 6. The tissue property distributions for each example are listed in Table 3. The clinical example is shown in Figure 7.

## 4 DISCUSSION

### 4.1 Surrogate models

The nonzero mean we observed in the error distribution of the OLS model may seem like an unusual outcome for an OLS model, but we note that mean error is only nonzero at specific insertion depths: When the error is averaged across all insertion depths, the mean is close to zero as expected. The asymmetry of the error distribution demonstrates that the normally distributed error assumption of OLS is violated for this problem. We believe this occurs because the relationship we are modeling is nonlinear. The creation of a ML surrogate could be accomplished by training a model to predict the shape of the needle at the next insertion step, similar to the system model used in state estimation models. This approach would be similar to “rolling out” the system model used with state estimation with no measurement updates. We trained models along these lines, namely OLS, lasso regression, ridge regression, and a MLP models. All of these models were unreliable—they would often reach the deepest insertion step with a large (greater than 10 mm) error, or would fail completely by predicting such a large deflection that the next step of the prediction would yield an overflow. We believe that the autoregressive paradigm does not work in this case due to the problem being nonstationary, that is, the statistical properties of the dataset change as the insertion depth increases. For this reason, we create a nonautoregressive model that predicts the deflection for an insertion of any depth.

Spectral bias in neural networks, such as MLPs, impedes them from capturing input features that vary across multiple length scales. We observe in our mechanics‐based model that tissue stiffness in a range of 0.001 to 0.2 MPa produce a realistic range of responses, so the input features vary by a factor of 200. We believe that this feature size range explains why our FFNN produces predictions with lower error than the MLP.

If a ML model trained on a mechanics‐based model is used for procedure planning, the needle placement error will be a function of both error introduced by the ML model and error introduced by the mechanics‐based model. The mean absolute error of 0.34 mm achieved by the FFNN is smaller than both the 3‐mm error assumption for outperforming a 12‐core systematic biopsy established by Robertson et al.,^5^ and the resulting 2.1‐mm assumption for targeting a subregion 50% by volume of a 3‐mm diameter tumor foci.^6^ As the error introduced by the ML model was much smaller than the level of error necessary for successful targeted prostate biopsies, the error introduced by the mechanics‐based model would determine if the needle placement error allows for successful procedure planning. Unfortunately, we cannot yet evaluate the needle placement error under clinical conditions due to difficulties in determining tissue properties in patients.

Regarding the widening distribution of error of the FFNN as shown in Figures 4a and 4b, we suspect that the increased number of layers increases the complexity of the relationship between the model inputs and outputs. As the capacity of the surrogate to model this complexity has limits, the combination of increasing depth and increasing number of layers leads to a nonlinear increase in error. Regarding the multimodality of the error distribution observed in Figure 4, the nonsmooth distribution of deflections in the dataset generated by the mechanics‐based model suggests that the multimodal error distribution is due to bias in the training set. The bias towards certain frequencies introduced by the scaling parameter of the Fourier feature transform may further bias the model toward predicting certain needle shape distributions over others.

Tancik et al. demonstrated that FFNN models can make predictions with lower error than MLP models on some datasets as MLP models have a spectral bias that diminishes their ability to fit high‐frequency data. We believe that the lower error and decreased bias observed in the FFNN are indicative that the MLP exhibits spectral bias on this dataset.

The neural network model was several orders of magnitude faster than the mechanics‐based model. While the mechanics‐based model ran at about 50 predictions per second on a single core of an AMD EPYC 7713p (corresponding to 2 s for a 100‐mm insertion with 1‐mm steps), the neural network inference ran at up to 20 million predictions per second on an Nvidia RTX 3090. However, the neural network requires batching, that is, running multiple inferences in parallel, to achieve the highest possible number of predictions per second. This high prediction rate makes the neural network model more practical to use for uncertainty quantification.

**FIGURE 8:**
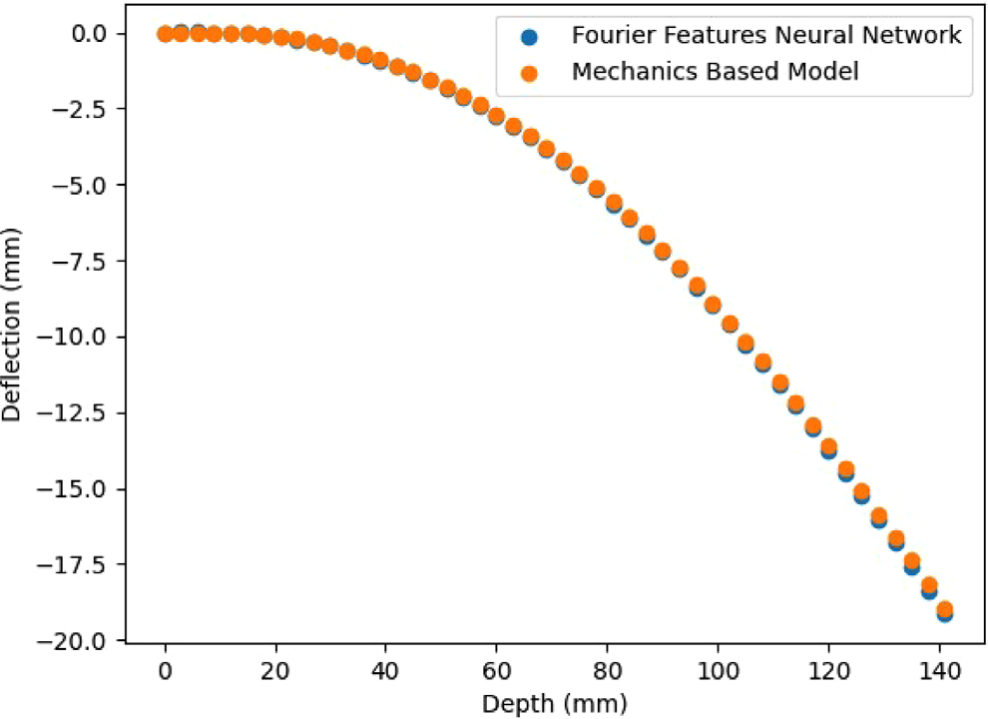
Example of Fourier feature neural network output compared to output of the mechanics‐based model for corresponding input.

We note that the FFNN has a small amount of noise in its output. While this is unrealistic as the shape of a real needle will be smooth, the amount of noise is small, as seen in Figure 8, and we do not believe that this amount of noise poses a problem for our uncertainty quantification model.

### 4.2 Experimental validation

Our mechanics‐based model and FFNN model are able to produce predictions that closely follow experimental needle deflections while using reasonable material properties. Unlike our model, which uses a linear elastic material model, Wang et al. use a one‐term hyperelastic Ogden model to model tissue in their mechanics‐based model. Wang et al. use an initial thickness, ti, of 20 mm in their Ogden model for all simulations. To make a direct comparison between our model and Wang et al.'s model, we need to account for the differences between the one‐term Ogden model and the linear elastic model. Unfortunately, Wang et al.'s model obtained material property parameters corresponding to elastic moduli much higher than published measurements. For example, Kelly et al. find a mean elastic modulus of 17.5 kPa for benign prostate tissue, and 70 kPa for cancerous prostate tissue in their review of the literature,^9^ while some of Wang et al.'s parameters give a tangent modulus over 10 MPa at no compression. Our model gives results for elastic moduli that align with those reported by Kelly et al. and others.

### 4.3 Uncertainty quantification model

Based on the changes in deflection distributions for varied positions of tissue layers shown in Figure 6, we observed that layers of stiff tissue early in the needle's path constrain the uncertainty of the final tip position, even if the needle subsequently passes through a layer of high uncertainty. We also observed that uncertainty in the final tip position is increased if a highly uncertain layer is placed early in the needle's path. Our interpretation of these results is that at shallow insertion depths, the needle receives minimal support from the tissue, thereby requiring less force to deflect. As the needle reaches a higher depth, more of its length is supported by tissue which resists further deformation. It is also intuitive that stiff layers of tissue at a low depth reduce the variance of possible needle paths since stiff layers of tissue support the needle more than less stiff tissue.

Most of the 10–20‐s runtime is spent on the Markov Chain sampling step where the tissue property distribution is sampled sequentially. The example shown in this work, where the distribution for each tissue property is independent, could have been performed much faster with direct sampling, where all of the samples can be collected in parallel. We believe it is plausible that tissue property distributions are nonindependent in each patient, which would be easier to model with Markov Chain sampling. However, modeling a nonindependent distribution would only be useful if it is accurate, and measuring this distribution based on biopsy procedures performed in many patients would be challenging. Furthermore, as it is unknown how tissue properties are distributed or correlated in patients, it is unknown how important modeling‐dependent distributions is and whether sampling the distribution using Markov Chain sampling would necessitate a long runtime or not.

The model's ability to predict needle trajectory in tissue enables physicians to proactively counteract needle deviation by adjusting the needle entry point, thereby achieving superior needle placement accuracy. In clinical practice, tissue stiffness parameters can be obtained either intraoperatively through ultrasound elastography or preoperatively via MRI‐based methods, then incorporated into the model for real‐time needle deviation prediction. For preoperative approaches, MR elastography or tomoelastography can provide the necessary tissue property data. Integration of MR‐derived stiffness information with intraoperative ultrasound imaging is readily achievable, thanks to the established MR‐TRUS fusion technology already in routine clinical use for target localization in prostate biopsies.

Much work still remains to create a model that captures enough phenomena relevant to needle deflection to be capable enough for clinical use. First, there are several features not currently captured in our ML model—anisotropic tissues, such as muscle tissue, as well as forces applied to the template to steer the needle, and rotation of the needle tip. Our mechanics‐based model is capable of modeling all of these features. However, we did not include these more complex elements in our dataset as it is challenging to experimentally validate such a model. Our mechanics‐based model is designed to model needle deflection in three dimensions, but the dataset we generated for our surrogate model only includes two dimension examples. All of these features have been incorporated into published mechanics‐based models, but no single published model contains all of these features. Once a mechanics‐based model capturing all of these features is created, a ML surrogate similar to one presented in this paper could be trained and could derive uncertainty quantification estimates. Second, in order to capture the orientation of muscle tissue, the anatomy of each patient must be accurately modeled. This would involve creating a generic anatomical model that includes the orientation of any anisotropic tissues and registering each patient to this model. Finally, realistic priors for tissue mechanical properties must be established. Some information about mechanical properties may be drawn from the literature as we have done in this work, but data from procedures would also be needed, which would involve recording needle insertions into patients and solving an optimization problem with the measured needle shapes and tissue structures.

We also note that the review from Kelly et al.^9^ appears to show multiple modes in the distribution of prostate elastic modulus (cancerous and benign, and possibly multiple modes observed from cancerous prostates) as well as a nonsymmetric distribution. However, it is not clear if these observations result from cancer state or from other variables such as age, treatment, or experimental protocols. If necessary, a more complex distribution could be accommodated in a future version of our model.

## 5 CONCLUSION

We have demonstrated an ML‐accelerated model of biopsy needle deflection that is several orders of magnitude faster than mechanics‐based models, paving the way toward incorporating uncertainty quantification into biopsy procedure planning. While there is still much work that would need to be completed for this method to be used in a clinical setting, this method shows promise for future applications in procedure planning for prostate biopsies as well as other transperineal procedures conducted with flexible needles such as cryoablation and brachytherapy.

## CONFLICT OF INTEREST STATEMENT

The authors declare no conflicts of interest.

## Data Availability

The code for the mechanics‐based model, the FFNN regressor, and our datasets are available on our github https://github.com/njhoffman11/needle_deflection_UQ.

## References

[1] Borghesi M , Ahmed H , Nam R , et al. Complications after systematic, random, and image‐guided prostate biopsy. Eur Urol. 2017;71(3):353‐365. 27543165 10.1016/j.eururo.2016.08.004

[2] Siddiqui MM , Rais‐Bahrami S , Truong H , et al. Magnetic resonance imaging/ultrasound–fusion biopsy significantly upgrades prostate cancer versus systematic 12‐core transrectal ultrasound biopsy. Eur Urol. 2013;64(5):713‐719. 23787357 10.1016/j.eururo.2013.05.059 PMC6301057

[3] Eklund M , Jäderling F , Discacciati A , et al. MRI‐targeted or standard biopsy in prostate cancer screening. N Engl J Med. 2021;385(10):908‐920. 34237810 10.1056/NEJMoa2100852

[4] Ahdoot M , Wilbur AR , Reese SE , et al. MRI‐targeted, systematic, and combined biopsy for prostate cancer diagnosis. N Engl J Med. 2020;382(10):917‐928. 32130814 10.1056/NEJMoa1910038 PMC7323919

[5] Robertson NL , Hu Y , Ahmed HU , Freeman A , Barratt D , Emberton M . Prostate cancer risk inflation as a consequence of image‐targeted biopsy of the prostate: a computer simulation study. Eur Urol. 2014;65(3):628‐634. 23312572 10.1016/j.eururo.2012.12.057 PMC3925797

[6] Tsourlakis M‐C , Stender A , Quaas A , et al. Heterogeneity of ERG expression in prostate cancer: a large section mapping study of entire prostatectomy specimens from 125 patients. BMC Cancer. 2016;16(1):641. 27530104 10.1186/s12885-016-2674-6 PMC4988055

[7] European Association of Eurology. EAU Guidelines on Prostate Cancer. Accessed Febuary 2 2025. https://uroweb.org/guidelines/prostate-cancer

[8] Gereta S , Hung M , Alexanderani MK , Robinson BD , Hu JC . Evaluating the learning curve for in‐office freehand cognitive fusion transperineal prostate biopsy. Urology. 2023;181:31‐37. 37579853 10.1016/j.urology.2023.08.005 PMC11363349

[9] Kelly NP , Flood HD , Hoey DA , et al. Direct mechanical characterization of prostate tissue—a systematic review. Prostate. 2019;79(2):115‐125. 30225866 10.1002/pros.23718

[10] Wang X , Wang J , Liu Y , et al. Alterations in mechanical properties are associated with prostate cancer progression. Med Oncol. 2014;31(3):876. 24504844 10.1007/s12032-014-0876-9

[11] Leung S , Zheng Y , Choi CYK , et al. Quantitative measurement of post‐irradiation neck fibrosis based on the young modulus: description of a new method and clinical results. Cancer. 2002;95(3):656‐662. 12209759 10.1002/cncr.10700

[12] Wang Y , Kwok K‐W , Cleary K , Taylor RH , Iordachita I . Flexible needle bending model for spinal injection procedures. IEEE Rob Autom Lett. 2023;8(3):1343‐1350. 10.1109/lra.2023.3239310 PMC10448781 37637101

[13] Webster III RJ , Kim JS , Cowan NJ , Chirikjian GS , Okamura AM . Nonholonomic modeling of needle steering. Int J Rob Res. 2006;25(5‐6):509‐525.

[14] Lehmann T , Rossa C , Sloboda R , Usmani N , Tavakoli M . Needle path control during insertion in soft tissue using a force‐sensor‐based deflection estimator. In: 2016 IEEE International Conference on Advanced Intelligent Mechatronics (AIM) . IEEE; 2016:1174‐1179.

[15] Fallahi B , Khadem M , Rossa C , Sloboda R , Usmani N , Tavakoli M . Extended bicycle model for needle steering in soft tissue. In: 2015 IEEE/RSJ International Conference on Intelligent Robots and Systems (IROS) . IEEE; 2015:4375‐4380.

[16] Roesthuis RJ , Abayazid M , Misra S . Mechanics‐based model for predicting in‐plane needle deflection with multiple bends. In: 2012 4th IEEE RAS & EMBS International Conference on Biomedical Robotics and Biomechatronics (BioRob) . 2012; IEEE; 69‐74.

[17] Quagliato L , Ryu SC . FEA modeling of soft tissue interaction for active needles with a rotational tip joint. IEEE Access. 2022;10:46291‐46301.

[18] Terzano M , Dini D , Rodriguez y Baena F , Spagnoli A , Oldfield M . An adaptive finite element model for steerable needles. Biomech Model Mechanobiol. 2020;19(5):1809‐1825. 32152795 10.1007/s10237-020-01310-x PMC7502456

[19] Yamaguchi S , Tsutsui K , Satake K , Morikawa S , Shirai Y , Tanaka HT . Dynamic analysis of a needle insertion for soft materials: arbitrary Lagrangian Eulerian‐based three‐dimensional finite element analysis. Comput Biol Med. 2014;53:42‐47. 25127407 10.1016/j.compbiomed.2014.07.012

[20] Li AD , Plott J , Chen L , Montgomery JS , Shih A . Needle deflection and tissue sampling length in needle biopsy. J Mech Behav Biomed Mater. 2020;104:103632. 32174391 10.1016/j.jmbbm.2020.103632 PMC7078072

[21] Liu W , Yang Z , Fang P , Jiang S . Deflection simulation for a needle adjusted by the insertion orientation angle and axial rotation during insertion in the muscle‐contained double‐layered tissue. Med Biol Eng Comput. 2020;58(10):2291‐2304. 32700291 10.1007/s11517-020-02212-x

[22] Singhal D , Narayanamurthy V . Large and small deflection analysis of a cantilever beam. J Inst Eng (India): Ser A. 2019;100(1):83‐96.

[23] Liu W , Yang Z , Jiang S . A mechanics‐based model for simulating the needle deflection in transverse isotropic tissue for a percutaneous puncture. J Mech Med Biol. 2019;19(6):1950060.

[24] Khadem M , Fallahi B , Rossa C , Sloboda RS , Usmani N , Tavakoli M . A mechanics‐based model for simulation and control of flexible needle insertion in soft tissue. In: 2015 IEEE International Conference on Robotics and Automation (ICRA) . IEEE; 2264‐2269.

[25] Jiang S , Wang X . Mechanics‐based interactive modeling for medical flexible needle insertion in consideration of nonlinear factors. J Comput Nonlinear Dyn. 2016;11(1):011004.

[26] Asadian A , Kermani MR , Patel RV . An analytical model for deflection of flexible needles during needle insertion. In: 2011 IEEE/RSJ International Conference on Intelligent Robots and Systems . IEEE; 2011:2551‐2556.

[27] Lee H , Kim J . Estimation of flexible needle deflection in layered soft tissues with different elastic moduli. Med Biol Eng Comput. 2014;52:729‐740. 25008003 10.1007/s11517-014-1173-7

[28] Behera B , Orlando MF , Anand RS . Prediction of bevel‐tip needle deflection using CART based decision tree. In: 2024 Third International Conference on Power, Control and Computing Technologies (ICPC2T) . 2024;IEEE; 769‐774.

[29] Tancik M , Srinivasan P , Mildenhall B , et al. Fourier features let networks learn high frequency functions in low dimensional domains. Adv Neural Inf Process Syst. 2020;33:7537‐7547.

[30] Craig. JWock82/pynite. November 3, 2017. T16:11:07Z.

[31] PyMC‐Devs. Pymc. November 2024.

[32] Wang Y , Al‐Zogbi L , Liu G , et al. Bevel‐tip needle deflection modeling, simulation, validation in multi‐layer tissues. In: 2024 IEEE International Conference on Robotics and Automation (ICRA) . IEEE; 2024:11598‐11604. 10.1109/icra57147.2024.10610110 PMC11494283 39439443

[33] Mo F , Zheng Z , Zhang H , Li G , Yang Z , Sun D . In vitro compressive properties of skeletal muscles and inverse finite element analysis: comparison of human versus animals. J Biomech. 2020;109:109916. 32807316 10.1016/j.jbiomech.2020.109916

[34] Pedregosa F , Varoquaux G , Gramfort A , et al. Scikit‐learn: machine learning in Python. J Mach Learn Res. 2011;12:2825‐2830.

[35] Ansel J , Yang E , He H , et al. PyTorch 2: faster machine learning through dynamic Python bytecode transformation and graph compilation. In: 29th ACM International Conference on Architectural Support for Programming Languages and Operating Systems (ASPLOS'24) . Vol 2. ACM; 2024.

[36] Moreira P , Patel N , Wartenberg M , et al. Evaluation of robot‐assisted MRI‐guided prostate biopsy: needle path analysis during clinical trials. Phys Med Biol. 2018;63(20):20NT02. 10.1088/1361-6560/aae214 PMC6198326 30226214

[37] Li M , Guo J , Hu P , et al. Tomoelastography based on multifrequency MR elastography for prostate cancer detection: comparison with multiparametric MRI. Radiology. 2021;299(2):362‐370. 33687285 10.1148/radiol.2021201852

[38] Sigrist RM , Liau J , Kaffas AE , Chammas MC , Willmann JK . Ultrasound elastography: Review of techniques and clinical applications. Theranostics. 2017;7(5):1303‐1329. 28435467 10.7150/thno.18650 PMC5399595

